# German translation of the PROMIS^®^ pediatric anxiety, anger, depressive symptoms, fatigue, pain interference and peer relationships item banks

**DOI:** 10.1186/s41687-023-00548-0

**Published:** 2023-02-21

**Authors:** J. Devine, A. Kaman, T. L. Seum, F. Zoellner, M. Dabs, V. Ottova-Jordan, L. K. Schlepper, A.-C. Haller, S. Topf, M. Boecker, J. Schuchard, C. B. Forrest, Ulrike Ravens-Sieberer

**Affiliations:** 1 Department of Child and Adolescent Psychiatry, Psychotherapy, and Psychosomatics, University Medical Center Hamburg-Eppendorf, Martinistr. 52, 20246 Hamburg, Germany; 2 Child Neuropsychology Section, Department of Child and Adolescent Psychiatry, University Hospital Aachen, Aachen, Germany; 3 Applied Clinical Research Center, Children’s Hospital of Philadelphia, Roberts Center for Pediatric Research, 2716 South Street, Philadelphia, PA 19146 USA

**Keywords:** PROMIS, Pediatric health, Translation, German, Anxiety, Anger, Depression, Fatigue, Pain, Peer

## Abstract

**Background:**

The present study aimed at the translation and cross-cultural adaptation of six PROMIS^®^ pediatric self- and proxy- item banks and short forms to universal German: anxiety (ANX), anger (ANG), depressive symptoms (DEP), Fatigue (FAT), pain interference (P) and peer relationships (PR).

**Methods:**

Using standardized methodology approved by the PROMIS Statistical Center and in line with recommendations of the International Society for Pharmacoeconomics and Outcomes Research (ISPOR) PRO Translation Task Force, two translators for each German-speaking country (Germany, Austria, and Switzerland) commented on and rated the translation difficulty and provided forward translations, followed by a review and reconciliation phase. An independent translator performed back translations, which were reviewed and harmonized. The items were tested in cognitive interviews with 58 children and adolescents from Germany (16), Austria (22), and Switzerland (20) for the self-report and 42 parents and other caregivers (Germany (12), Austria (17), and Switzerland (13)) for the proxy-report.

**Results:**

Translators rated the translation difficulty of most items (95%) as easy or feasible. Pretesting showed that items of the universal German version were understood as they were intended, as only 14 out of 82 items of the self-report and 15 out of 82 items of the proxy-report versions required minor rewording. However, on average German translators rated the items more difficult to translate (M = 1.5, SD = 0.20) than the Austrian (M = 1.3, SD = 0.16) and the Swiss translators (M = 1.2, SD = 0.14) on a three-point Likert scale.

**Conclusions:**

The translated German short forms are ready for use by researchers and clinicians (https://www.healthmeasures.net/search-view-measures).

## Plain English summary

A multitude of questionnaires exist, which are not comparable due to different questions or no available translations. PROMIS is an initiative, which was funded by the National Institute of Health in the US, to build better, i.e., more precise and efficient questionnaires, which can be used and compared worldwide. The PROMIS questionnaires include paper-and-pencil short forms and computerized adaptive tests. So far numerous PROMIS surveys have been created using advanced methodologies. They can be used by health care professionals to assess different aspects of health and compare the results internationally. To allow for international comparability of studies using those questionnaires, they need to be translated. This study reports the thorough translation process of the US-American PROMIS^®^ questionnaires measuring anxiety, anger, depressive symptoms, fatigue, pain interference, and peer relationships in children and adolescents into German. The translation included researchers, children, and parents from Germany, Austria, and Switzerland to ensure that the final German version is fully and equally well understood in all of those German-speaking countries. The article describes the translation process, so that the user can understand the translations and use them in an informed way. The translated German questionnaires are ready for use by researchers and clinicians. (https://www.healthmeasures.net/search-view-measures).

## Background

In 2004, the NIH launched a program of research called the Patient Reported Outcome Measurement Information System (PROMIS^®^) [1]. The goal of PROMIS was to provide clinicians and researchers access to short, precise, valid, and responsive adult- and child-reported measures of health (for more information see www.healthmeasures.net; for pediatric applications see [2]) via advanced measurement methods (Item Response Theory – IRT [3]) as well as computerized adaptive testing (CAT [4]). PROMIS was carried out by a network of research and health care centers across the US with collaborations worldwide aiming at creating standardized questionnaires, which can be used for national and international comparisons of clinician’s and study results [5, 6]. It provides a conceptualization of health for adults and children and developed, validated, and translated item banks for short and long fixed-length forms and CATs. The advantage of utilizing IRT methods and CATs is that item banks can be established from which either a CAT or a specific short-form can be drawn, thus minimizing the number of items displayed to the child while maintaining measurement precision and responsiveness [7–10]. Since 2013 there has been a steady increase in the translation of PROMIS measures including into Chinese [11, 12], Dutch-Flemish [13, 14], Spanish, German [15–17], Nepali [18] and other languages.

**Fig. 1:**
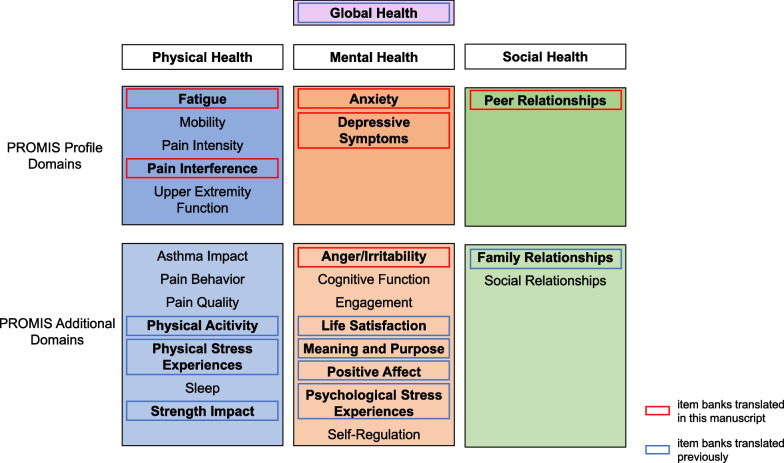
PROMIS Conceptualization of pediatric health

The PROMIS conceptual framework of pediatric health subsumes the domains of physical, mental, and social health under the general concept of global health. Each domain comprises a set of subdomains such as mobility, fatigue, and pain (physical health), anxiety, depressive symptoms, and life satisfaction (mental health) or family and peer relationships (social health; see Fig. 1).

Due to our interest in the above mentioned methodological potentials and our experience in developing pediatric health-related quality of life (HRQoL) measures such as the KIDSCREEN [﻿^20^] and the German Kids-CAT [19], we first focused on developing and empirically testing the PROMIS pediatric subjective well-being domains (SWB) including the subdomains positive affect, life satisfaction, meaning, and purpose [21–23]. Since 2012 our team has also carried out the first large-scale translation of PROMIS pediatric item banks to German (and Spanish) to improve international comparability of health screenings of children and adolescents [15]. We first translated and cross-culturally adapted eight PROMIS pediatric item banks for use in the BELLA study [24], which extended beyond SWB to physical activity, experience of stress, and family relations ([15, 16], see Fig. 1 with previously translated item banks framed in blue). Subsequently, we translated six further pediatric PROMIS item banks (see Fig. 1 item banks framed in red) to German, which is the focus of this article. Specifically, we translated the item banks (version 1) and short forms (version 2) of the PROMIS pediatric subdomains anger (ANG, [25]), anxiety (ANX, [26]), depressive symptoms (DEP, [26]), fatigue (FAT, [27]), pain interference (P, [28, 29]), and peer relationships (PR, [30]) both as self-report and proxy-versions [31], to a universal German version. The objective was to attain semantically and linguistically cross-cultural as well as content- and conceptually equivalent translated item banks and short forms, which can be used in Germany, Austria, Switzerland, and other German-speaking countries like Luxembourg for example.

## Methods

### Translation approach

A total of 164 items with 82 self and 82 proxy item versions were translated: ANG (5 items), ANX (13 items), DEP (13 items), FAT (23 items), PAIN (13 items), and PR (15 items), plus 3 divergent items of proxy versions (for ANG and DEP, FAT), and a Likert scale with 5 response options. Higher scores on those PROMIS items correspond to higher levels of the concept name (e.g., greater fatigue or better peer relationships). We translated the item banks to be used with German-speaking children in Germany, Austria, and Switzerland (one universal German version). To account for variations in wording between countries using the same language, our translation team was comprised of two native translators from each country (six translators in total), who were external and recruited for the purpose of this project. In addition to those translators, two experts acted as translation process managers (TPM), who oversaw and managed the process (see Appendix 1).

**Fig. 2:**
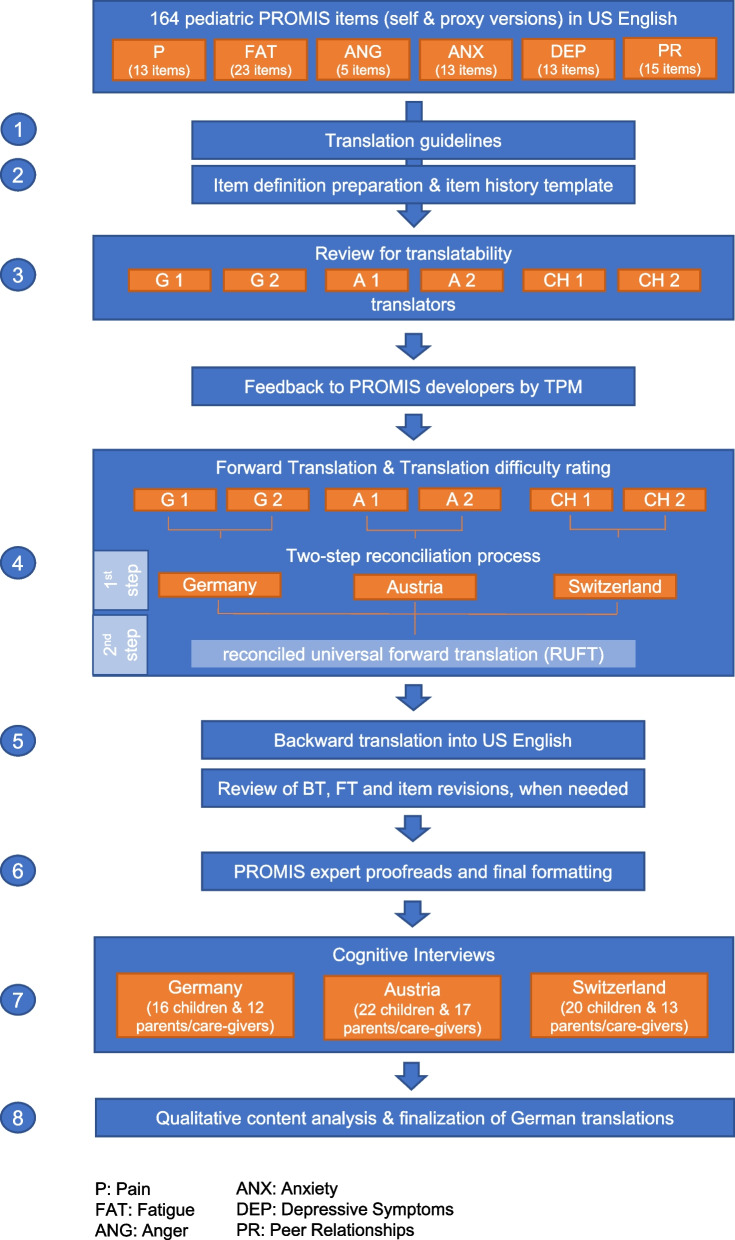
FACIT translation methodology

We applied the “universal translation approach” following a widely used, multi-step forward–backward translation technique with cognitive interviews complying to the recommendations of the FACIT translation methodology (see Fig. 2 [32, 33]), which is the current PROMIS translation standard and in line with the International Society for Pharmacoeconomics and Outcomes Research (ISPOR) PRO Translation Task Force [34, 35] and PRO Consortium recommendations [36].

The same methodology was applied to the translation of 8 PROMIS pediatric item banks in 2018 [16] as well as to other pediatric translations processes [11, 13] and may be the best choice as long as no gold standard of translation exists [37].

### Translation guidelines

First, translation guidelines were written based on a review of the literature, translation guidelines, and PROMIS standards (see Protocol, Appendix 2). Items were to be translated as accurately as possible, keeping the intended item definition/content in mind and producing easy, child-friendly item translations avoiding region-specific or colloquial words.

### Original items

Then PROMIS provided the original item versions and conceptual and technical item definitions, based on which a comprehensive item history template was developed to guide the translators, facilitate accurate translation, enable thorough documentation, and ensure conceptual/cross-cultural equivalence (see Table 1 as an example).

**Table 1:** Exemplary item definitions provided by the U.S. authors and forward translation template developed for the translation process

	Item	English original			Definitions		Forward translation			
							G1	Daniel Bullinger	Rating translation difficulty 1 = easy to translate 2 = feasible to translate 3 = difficult to translate	Date
							G2	Anne-Catherine Haller		
	ID	Context	Stem	Response options	Concept	Technical	Forward translation: stem		Translation difficulty	Comments
*Peer relationships (Self)*										
1	5018R1	In the past 7 days,	I felt accepted by other kids my age.	Never; Almost never; Sometimes; Often; Almost always	The idea of this item is to ask if the patient feels that other children the same age accept and approve of him/her as one of their own	*To feel accepted*: to feel approved or recognized; to feel “like a member of the group”	G1			
							G2			
2	5058R1	In the past 7 days,	I was able to count on my friends.	Never; Almost never; Sometimes; Often; Almost always	The idea of this item is to ask if the patient felt that he/she could depend or rely on his/her friends	*To count on*: to depend or to rely on	G1			
							G2			
3	5056R1	In the past 7 days,	I was able to talk about everything with my friends.	Never; Almost never; Sometimes; Often; Almost always	The idea of this item is to ask if the patient was capable of talking about all matters with his/her friends	*To be able*: to have the ability; can; to be capable *Everything*: all matters; everything or particular of an aggregate or total; all *Friend*: a person other than family, whom one knows, likes, and trusts	G1			
							G2			
4	1147R1	In the past 7 days,	I was good at making friends.	Never; Almost never; Sometimes; Often; Almost always	The idea of this item is to ask if the patient was skilled at acquiring new friends	*To be good at*: to be skilled at; to employ successful strategies for *To make friends*: to acquire new friends; to become friends with other people	G1			
							G2			

### Item translation

The TPMs informed the translators about the translation process, provided the items, and instructed the translators. Two independent native translators specializing in pediatric research from each country (Germany, Austria, Switzerland) were asked to review the items for translatability, that is to comment on the translation difficulty issues such as unclear idioms, ambiguous words, unclear concepts, etc. The TPMs summarized those comments and reported them back to the PROMIS test developers to clarify and/or improve the item definitions, if needed, so that concept validity was ensured. Subsequently, the items were forward translated (FTs) to German. In each country, translators worked independently from one another following the translation guidelines (Appendix 2) to create the translations. They rated the translation difficulty of the items using a three-point Likert scale (1: easy, 2: feasible, and 3: difficult to translate).

### Reconciliation process

A two-step reconciliation process was conducted: The translators first reconciled their versions within each country agreeing upon a good version of the FT. Then all translators and experts from each country held a teleconference to harmonize the versions to a universal German version, the final product of which was a Reconciled Universal Forward translation (RUFT).

### Back translation and consented universal translation

The German versions were back translated (BT) by an independent professional translator not involved previously, who had no knowledge of the original English source items or the item definitions. An expert group consisting of the back translator, forward translators and the TPMs reviewed the BTs and the FTs, and the translations were revised where necessary, based on the equivalence between BTs and the source versions. In this way a consented universal translation (CUT) was created. The CUTs underwent a quality review, were formatted, and proof-read by an independent PROMIS translation expert.

### Cognitive interviews

Cognitive Interviews (CIs) were conducted in Germany, Austria, and Switzerland to ensure the feasibility, relevance, and equivalence in comprehension of the CUT [32, 38–40]. Prior to that, ethical approval for the translation projects had been given by the Ethics Committee of the Chamber of Psychotherapists (Psychotherapeutenkammer) of Hamburg, Germany. For the CIs, children from the general population, residing in the specific countries (Germany, Austria, Switzerland) were included under the condition that they were able to speak and read German and had no mental or physical condition that interfered with their ability to be interviewed. Children and parents/caregivers were recruited and informed about the study via email and gave their informed consent before participation. CIs of the self-report items were performed with n = 58 children and adolescents aged 8–17 years (Germany: n = 16, Austria: n = 22, Switzerland: n = 20). For practical reasons, focus groups with 2–8 participants were chosen for the cognitive debriefing in Austria (Vienna/Linz) and Switzerland (Bern), whereas in Germany (Hamburg) participants were mainly interviewed individually and focus groups were only conducted if siblings or close friends participated. CIs were performed by trained staff.

To ensure that parents found the items easy to understand and understood them the same way as intended, CIs were also performed with n = 42 parents or other caregivers (of 8–17-year-old children/adolescents; Germany: n = 12, Austria: n = 17, Switzerland: n = 13).

The interviews lasted about 60 min and started with the interviewer giving the 32 children/adolescents and parents a questionnaire containing a selection of 25 PROMIS items and asking them to rate them on a three-point Likert scale (1 = difficult, 2 = potentially problematic, 3 = easy to understand). Then the interviewers asked questions about the cognitive understanding of the items using a mix of cognitive techniques, such as “general probing” and “paraphrasing” [40]. Interviews were recorded and pilot testing reports were written, which were fed back to the translators and TPM, in order for the items to be optimized when needed. Children and adolescents received a 10 Euro gift voucher for their participation.

The CI results were reviewed by the TPMs and feedback was compiled. The translation team then discussed the results and agreed on final changes based on the translation issues identified during the CIs. The final versions were finally reviewed again and approved by the PROMIS Statistical Center.

Note that the described steps were performed mainly for the self-report versions of the items, because the proxy versions have the exact same wordings except that the perspective is changed (i.e., “I” was replaced by the words “my child”).

## Results

### Translatability review

Using the three-point Likert scale, translators found most items “easy” to translate (69%), 26% of the items slightly more challenging, but “feasible to translate”, and 4% of the items were categorized as “difficult to translate”. For example, difficult items used ambiguous words (“feel mad” = could either mean “angry” or “insane”), idioms (“it hurt all over my body” = my whole body hurts) or unclear words, which led the translation manager to clarify their content with the US developers. This was also the case for the item “I felt upset” (ANG items), the item word “worried” (ANX item; the translation “anxious” was considered too strong a translation by the developers, instead preoccupied/concerned were suggested as translations) and the DEP items “I felt alone” versus “I felt lonely” (the first item implying that no one is physically present, the second being possible if others are present). The translation which communicated emotional loneliness best, regardless of physical presence was kept, i.e., “Ich fühlte mich einsam”.

In the FAT item bank, in 10 out of 23 items the translation expert raised an issue with the words “too tired (to…)”. This wording does not work well in German because using the literal translation “zu müde” in German implies that things cannot be done, whereas in English the items imply that things may still get done although with difficulty. However, to capture the English original content as much as possible the literal translation was kept after a thorough discussion.

In the PAIN item bank, the item “It was hard to have fun when I had pain” the TPM needed to ask the developers whether “have fun” referred to the feeling of enjoyment, or to participating in joyful activities (the former was the case).

**Fig. 3:**
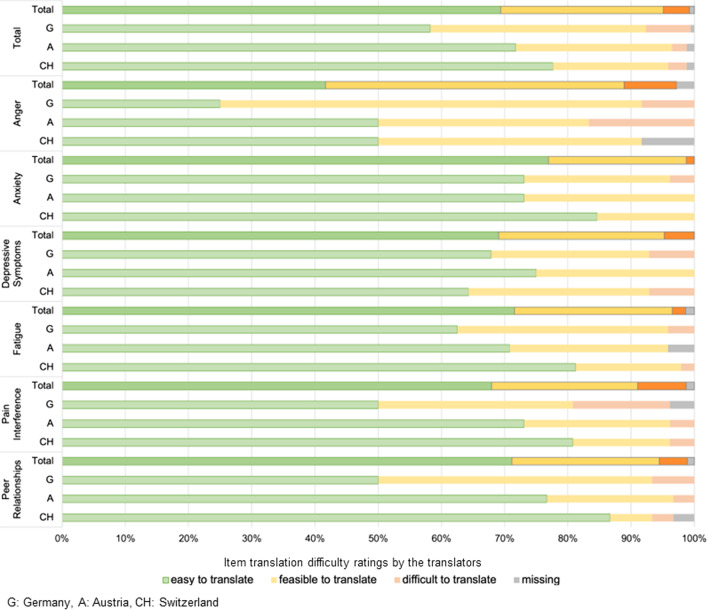
Translation difficulty ratings

Figure 3 summarizes the translation difficulty ratings across the item banks. On average German translators rated the items more difficult to translate (average = 1.5; standard deviation (SD) = 0.20) than the Austrian (average = 1.3; SD = 0.16) and the Swiss translators (average = 1.2; SD = 0.14).

The ANG item bank was the most difficult to translate due to item words such as “I felt fed up” where item definitions were multifaceted, covering feeling impatient, disgusted, and bored beyond endurance. The ANX item bank was rated the easiest to translate. Six out of 13 items used the word “worried”, which is easily translatable to “besorgt/sich Sorgen machen”, and items using words such as “scared or afraid” seemed easy to translate as there are similar anxiety words in German (Angst haben, fürchten, erschrecken).

For the DEP item bank, translation issues arose with the item “I didn’t care about anything”, because it is, combined with the response option “never”, a double negative. The items using the word “thing/s” posed some challenges to the translators, too (e.g., “I had trouble finishing things”, German: “Dinge”/ “Aufgaben”).

In the PAIN item bank, the word “run” and “(walk a) block” were difficult to translate, due to different possible translations of run: “laufen” (run fast), “gehen” (walk), “rennen” (sprint), “joggen” (jog), and due to the concept of a block not being a common distance measure in German. The latter was solved by describing “a block” as a short distance (“kurze Strecke (100 m))” as recommended by the test developers.

For the PR item bank, translation issues were that the item “I was good at making friends” was hard to translate as it originally captures how easy it is for someone to make new friends “in the last week”. The underlying American concept of making friends is different from the German concept; there, making friends is usually considered to be a process of several weeks instead of one week. In addition, there is no natural, literal good way of translating “I felt good about my friendships” in German. Translators finally decided on “habe ich mich mit meinen Freundschaften wohl gefühlt”, but this still does not sound very natural to a native German child. Further, the concept of “I (played alone and) kept to myself” was not that easy to translate to German, because translators doubted that young children understand the content easily. They finally decided upon “(Ich habe allein gespielt und) bin für mich selbst geblieben”.

### Reconciliation process

The reconciliation process took about 2.5 h and the translators and experts carefully discussed most items to ensure cross-cultural equivalence. Cross-cultural differences in wordings became apparent. For instance, the item “I felt fed up” was translated “Ich hatte die Nase voll” (literally: “my nose was full/I was fed up”) by German and Swiss translators, while the Austrian translators suggested “Ich war angefressen” (literal “I was eaten up/fed up/full”). The latter was not used, because in Swiss German it would have meant that the child is very interested in something.

Also, the word “angry/mad” was discussed among translators and translation options included “verärgert”, “wütend”, “sauer” (colloquial German) or “zornig” (preferred by Austrian translators). In the end, “wütend” as a clear moderate emotional word was agreed upon for two items and “zornig” for one item.

For some item banks, translators discussed that US items often included the word “*feeling*” (e.g., “I felt nervous”), while in German the natural equivalent is “being” (e.g., “I *was* nervous”). To improve item understanding for German children the word “felt” was replaced by “was”.

Also, in the reconciliation process it was found that the letter sharp “ß” is not used in Switzerland. It was suggested to create different language versions or write the word without the “ß” in brackets in universal items (e.g., “Ich habe mir Sorgen darüber gemacht, was mir zustoßen (zustossen) könnte”, “Es ist mir schwergefallen Spaß (Spass) zu haben”).

For the PR item bank, translators discussed whether words like “kids” were age appropriate for children and adolescents up to 17 years of age, and it was suggested to translate “kids” as “Kinder/Jugendliche” (i.e., add youths to the item). In addition, test developers were asked to reword the word “play” for adolescents, but in the end those items included other activities than playing that were age-appropriate for youths, so the word “play” remained as one activity next to the others in the item. Also, it was discussed that while it may take longer to make friends the older a child becomes (PR item “it was easy for me to make friends”), in times of digitalization the German concept of making “friends” may have changed as German children/adolescents tend to refer to peers as “friends” more quickly than a decade ago (when friends were only called friends after a very substantial amount of time). This issue could not be resolved in the translation process. Another age-related translation issue occurred with the item “other kids wanted to be with me”, of which the most common translation was “andere Kinder wollten mit mir zusammen sein”, however translators discussed that teenagers may misunderstand the phrase “zusammen sein” as “dating”, which would distort the meaning. Hence the universal translation “andere Kinder/Jugendliche wollten mit mir Zeit verbringen” (“other kids wanted to spend time with me”) was agreed upon.

### Back translation (BT)

The back translations showed some small wording differences. For instance, for the ANG bank, words like “(being) furious” and “annoyed” were used, which were not part of the original items, but fit the content. For anxiety, “anxious” was synonymously used as “scared” and “afraid”. For depression, the word “felt (sad)” in English was often replaced by the word “was (sad)” – replicating the issue discussed above. For FAT, the BT showed that the word “bed” had been mistakenly omitted from one item in the translation process and was again inserted to ensure literal equivalence. For PAIN, the BT showed that the duration of standing still was implicit in the original item version but needed to be specified in the translation to avoid misunderstandings. In total, the review of the BTs led to six minor changes in item wording.

### Cognitive interviews (CI)

Fifty-eight children and adolescents and 42 of their caregivers took part in the cognitive interviews (see Table 2).

**Table 2:** Age and gender of the sample, who underwent cognitive interviewing

Country	Children aged 8–11 years			Adolescents aged 12–17 years			Parents/care-givers		
	m	f	total	m	f	total	m	f	total
G	4 (44%)	5 (56%)	9	5 (71%)	2 (29%)	7	2 (17%)	10 (83%)	12
A	2 (20%)	8 (80%)	10	9 (75%)	3 (25%)	12	3 (18%)	14 (82%)	17
CH	5 (50%)	5 (50%)	10	6 (60%)	4 (40%)	10	2 (15%)	11 (85%)	13
Total	11 (38%)	18 (62%)	29	20 (69%)	9 (31%)	29	7 (17%)	35 (83%)	42

*M*, male, *f*, female, *G*, Germany, *A*, Austria, *CH*, Switzerland

The CIs took up to two hours, with parent interviews taking longer than those with younger children. Overall, the motivation of the children and parents was very high and children and parents liked the CI as the material displayed was perceived as child-friendly (e.g., with smileys etc.). The CIs showed detailed insights into the comprehension of the item words by children and parents. After the recorded sessions were summarized and reported to the TPM, the CI results led to minor revisions of seven items, which are displayed in Table 3.

**Table 3:** Results of the cognitive interviews, which were particularly interesting and resolved in the cross-cultural harmonization process

Domain	English original	German translation	CI issue	Resolution
*CI results, which led to item revisions*				
Anger	I felt mad	Ich war zornig	**Zornig** was not part of everyday language for some children	Translation was changed to “Ich war wütend”
Depression	I felt alone	Ich habe mich alleine gefühlt	During the CI it became obvious that the tense of the translation was not the same as in the original item	Translation was corrected to “Ich fühlte mich allein”
Depression	I felt lonely	Ich habe mich einsam gefühlt	See above	Translation was corrected to “Ich fühlte mich einsam”
Fatigue	I got tired easily	Ich bin leicht müde geworden	Children misunderstood “leicht “ (quickly) as being a little tired	Item was revised to “Ich bin **schnell** müde geworden “
Pain	I had trouble doing schoolwork when I had pain	Ich hatte Schwierigkeiten Schul- und Hausaufgaben zu machen, wenn ich Schmerzen hatte	Children and parents in all three countries did not differentiate well between “Schulaufgaben” (work in class) and “Hausaufgaben” (homework), they understood Schulaufgaben as Hausaufgaben	The concept “Schulaufgaben “ as “Aufgaben “ in der Schule (work at school/in class) was specified in the item translation to “Ich hatte Schwierigkeiten die **Aufgaben in der Schule und Hausaufgaben** zu machen “
Pain	It was hard for me to pay attention when I had pain	Es war schwer für mich aufzupassen, wenn ich Schmerzen hatte	Most of the Swiss kids understood "aufpassen (pay attention)" only relating to school. All kids in all three countries prefer "konzentrieren (concentrate)" rather than "aufpassen (pay attention)"	The translation was revised to “Es ist mir schwergefallen, mich zu **konzentrieren**, wenn ich Schmerzen hatte.“
Pain	It was hard to get along with other people when I had pain	Es ist mir schwergefallen, mit anderen auszukommen, wenn ich Schmerzen hatte	The item was understood well by all children, but nearly all participants would prefer the addition of the word "gut (well)" since this term is more common within German language	The translation was revised to „Es ist mir schwergefallen, **gut** mit anderen auszukommen, wenn ich Schmerzen hatte “
*CI results, which did not lead to item revisions*				
Pain	It was hard for my child to run when he/she had pain	Es ist meinem Kind schwergefallen, zu joggen oder zu rennen, wenn es Schmerzen hatte	Young children did not understand the word “joggen”	Because children understood the added translation of that word (”rennen”), the translation did not need to be revised
Pain	Walking a block	Eine kurze Strecke/100 m gehen	Children understood the phrase “kurze Strecke” (short distance), but not the culturally adjusted metric 100 m very well	Because children understood the added translation of the word (= kurze Strecke), the translation did not need to be revised
Anger	My child felt upset I felt upset	Ich habe mich aufgeregt	One Austrian mother stated that the item could be misunderstood positively as "excited", which is also a meaning of "aufgeregt" Children perceived the word “upset” ambiguous	The ambiguity had been fed back to the test developers, but to maintain the content, the translation was kept as it is
Peers	During the past 7 days, I was good at making friends	In den letzten 7 Tagen, ist es mir leicht gefallen Freundschaften zu schließen	Children discussed that it took longer than one week to make friends	The item was nevertheless kept to keep the intended content of the original item

## Discussion

The PROMIS pediatric self- and proxy report items of the item banks (version 1) and short forms (version 2) covering the subdomains anger, anxiety, depressive symptoms, fatigue, pain interference, and peer relationships were translated well to German (for exemplary items see Appendix 3). The translation process proved most items easy or feasible to translate and most items were understood as intended in pretesting. Only 14 out of 82 items of the self-report and 15 out of 82 items of the proxy report items required minor rewording during the translation process. Our study shows that it is important to avoid ambiguous and multifaceted words when developing a questionnaire because these words will create translational difficulties later on. In general, the translation of some ANG items proved to be the most difficult one as some of the items were multidimensional. For example, the item word “upset” evoked translation issues at several stages of the translation. These were similar to translation difficulties found in translations of this item to German by Jakob et al. ([17] in a depression measure) and to Spanish ([41] in the Neuro-QoL). Interestingly this item was also different in the psychometric analyses performed by Kaman et al. [42] in that it was the only item which did not show floor effects on the anger scale.

In addition, the study found cross-cultural differences in concepts, e.g., between the German and the US culture regarding the speed with which participants make friends, or regarding the comprehension of words “zornig” vs. “wütend” between the German, Austrian-German, and Swiss-German languages.

Further, we would like to point out that questionnaires should always keep the target participants in mind. We found that teenagers may not feel addressed when using words such as “children” in the instruction/item texts requiring us to add the word “adolescents” (even though this was not part of the original item text).

Similar to previous studies [12–14, 16], translating units of measurement/metrics (blocks, meters etc.) usually pose some challenges cross-culturally and were solved accordingly. Differential item functioning analyses are needed to explore whether cross-culturally different measurement units (e.g., metric vs. imperial measures; colloquial terms such as a “block”) may result in different item difficulties and may need language-specific item calibrations.

Further, similar to our previous study, the translation of some items needed two (synonymous) words to capture the intended concept in a way that all children/adolescents of varying ages and of all German-speaking countries could understand (see also [16] or [18]).

The strengths of this translation are its rigorous PROMIS translation methodology and the large sample size of particularly Swiss and Austrian children and parents interviewed in cognitive debriefings – exceeding the CI sample sizes of our previous translation project [16]. Limitations of this study were that many of the children and parents participating in the CIs were overall healthy and only age and gender was assessed for the participants. While this study shows very good comprehensibility of the translated items, it may be that children or parents with a lower socioeconomic or educational background, those coming from an ethnic minority background or those with chronic diseases or learning disabilities may have difficulties in understanding the items. This needs to be investigated in future studies with more heterogeneous samples. Further, the CIs did not include questions about the relevance of the items as indicators of the domains assessed in Germany. In addition, this study did not include psychometric analysis or norming of the translated items (for US norms of the PROMIS source measures see [43]), thus criterion and technical equivalence of the translations need to be checked by psychometric validation studies (e.g., [44] or [45]) to provide additional information of the degree of cross-cultural equivalence achieved in the present study.

In 2019 our research group performed the first psychometric analyses of a German PROMIS pediatric translation [42]. The translated pediatric anger item bank demonstrated good psychometric properties, including satisfactory distribution characteristics, unidimensionality, good internal consistency, and congruent validity. Also, German normative data for this item bank was established. In 2014–2017, the BELLA study administered the German PROMIS pediatric translations of the subjective and family well-being item banks, physical activity, and peer relationship item banks as well as the PROMIS Global Health scale [46], and for adolescents aged older than 18, the Profile 29 [47]. Thus, German representative data is now available for psychometric analysis and norming of the translated tools.

## Conclusion

Six pediatric PROMIS^®^ item banks (anxiety, anger, depressive symptoms, fatigue, pain interference and peer relationships) were successfully translated into German and adapted for use in Germany, Austria, and Switzerland with relatively minor changes to the content of the original English items. The translated item banks cover the items of the corresponding current PROMIS pediatric short forms (version 2) and also allow for computerized adaptive testing (CAT) for some of the translated dimensions (e.g., fatigue). Both the item banks and short forms are available at https://www.healthmeasures.net/search-view-measures. They are ready to be used in clinical studies and for pediatric public health research offering clinicians and researchers nationally and internationally cross-culturally comparable standardized assessments, which are of high psychometric quality.

## Data Availability

The manuscript does not contain any individual person’s data. Data is available at the University Medical Center Hamburg-Eppendorf, Department of Child and Adolescent Psychiatry, Psychotherapy, and Psychosomatics, Martinistr. 52, 20,246 Hamburg, Germany, Email: ravens-sieberer@uke.de.

## Appendix 1: Experts and Translators/Qualifications.

- Prof. Ravens-Sieberer:
  - Professor for Public Health, Health Psychology and Health Care Services Research.
  - Research director of the Department of Child and Adolescent Psychiatry, Psychotherapy, and Psychosomatics she is leading the research group “Child Public Health”.
  - International coordinator-in-chief of the European KIDSCREEN project that developed a cross-cultural applicable patient reported outcome measure for children and adolescents simultaneously in 13 European countries. Prof. Ravens-Sieberer led and supervised translations and cultural adaptations of the KIDSCREEN and the KINDL (another PRO instrument for children, which she developed).
- Dr. Veronika Ottová-Jordan, MPH,
  - Worked as Research Assistant in Child Public Health at the University Medical Center Hamburg-Eppendorf, Department of Child and Adolescent Psychiatry and Psychotherapy.
  - Has experience in managing international and national projects. Among the most important are the Health Behavior in School-aged Children (HBSC) study and the European RICHE project (RICHE—Research Into Child Health in Europe).
  - Has also been involved in several projects on the linguistic validation of health instruments and, in the HBSC study, was responsible for coordinating the German translations, which are conducted in cooperation with Child and Adolescent Health Research Unit (CAHRU), at the University of St. Andrews, Scotland.
- Laura Schlepper (TPM)
  - Researcher at Nuffield Trust as a Researcher since March 2018. She provided support on a range of quantitative and qualitative projects relating to health and social care.
  - Worked as Research Assistant in Child Public Health at the University Medical Center Hamburg-Eppendorf, Germany, where she assisted with a national survey on child and adolescent mental health and managed the translation and linguistic validation of paediatric patient-reported outcome measures.
  - Holds an MSc in Mental Health Service & Population Research from the Institute of Psychiatry, Psychology & Neuroscience, King’s College London, where her research focused on mental health-related stigma and discrimination, and a BSc in Psychology with Sociology from the University of Hull.
- Michaela Dabs (TPM)
  - Sociologist
  - Research assistant at the Department of Medical Psychology, University Medical Center Hamburg-Eppendorf
- Children’s Hospital of Philadelphia (CHOP) project lead: Christopher Forrest.
- Translation coordination (UKE): Laura Schlepper and Michaela Dabs
- Professional Translators:
  - German FT: Daniel Bullinger, Christiane Focking,
  - Austria FT: Sabine Topf, Christine Zenker,
  - Swiss FT: Mirjam Urfer, Jonas Müller
  - German BT: Ann Marie Bohan.

The PROMIS team worked closely with Helena Correia from the PROMIS Statistical Center throughout the translation process ensuring overall cross-cultural harmonization, and in the translatability review.

## Appendix 2: Translation and Review guidelines.

### Guidelines for forward translation (FT)

- Examine the item matrix or Excel file and see how it is organized. The Excel file is divided in seven spreadsheets you can select separately *(Response Options, ANG, ANX, DEP, FAT, PAIN, PEER).*
- Read each original English language item carefully.
- Read the concept definition to verify the intended meaning of the English item.
- Refer to the spreadsheet to see how the item stems connect with the recall period or another context, and with the answer options. You should ensure that your translation works with both the item stem and the response options.
- Use language that a child can understand.
- If you are unsure as to the most appropriate translation for an item, or feel there may be more than one suitable alternative, please feel free to provide those alternative formulations in the target language.
- Please make a note of any words, terms or phrases, which were particularly difficult to translate.
- Please provide conceptual translations (i.e. translations that capture the intent of the original but which are expressed as naturally as possible in the target language), rather than literal translations.
- Use universal language as much as possible, instead of country-specific terms.
- Avoid idiomatic expressions that could be misinterpreted, unless the source item also uses an idiom and the target language has one, which is equivalent.
- Provide gender-inclusive wording where applicable (for example, “friendly” or “co-operative” instead of “brotherly”).
- Stay true to the intended meaning of the source (no additions or subtractions) and to the format (e.g., underlining, bolding, punctuation).
- Verify that the same terms are translated consistently throughout the questionnaire or item bank, unless the item definitions specify that they have different meanings.
- Please begin with the forward translation of the response options:
  1. Enter the translation for each of the response options in the appropriate row under column ‘*Forward translation’*.
  2. You will find labeled rows for each of the translators (e.g., *G1, G2*).
  3. Please rate the translation difficulty (1 = easy, 2 = feasible, 3 = difficult).
  4. Comments regarding the forward translation can be entered in column ‘*Comments’*.
- Please go on with the forward translation of the item stems (spreadsheets *ANG, ANX, DEP, FAT, PAIN, PEER*):
  1. Enter the translation for each of the item stems in column *‘Forward translation: stem’*—please only translate the item stem, not the context.
  2. You will find labeled rows for each of the translators (e.g., *G1, G2*).
  3. Please rate the translation difficulty (1 = easy, 2 = feasible, 3 = difficult).
  4. Comments regarding the forward translation can be entered in column ‘*Comments’*.
- When you have translated all items, please review them to ensure consistency of style and naturalness of language.

All of the above criteria apply to any translator or reviewer when providing forward translations into the target language (i.e., during reconciliation, review process, and finalization of the translation).

### Guidelines for the reconciliation of the forward translations from English into target language by the translators

- Review the forward translations for each item.
- Select one of the forward translations for each item or provide a new option; this could be a modified version of one of the forward translations, a hybrid version, or a new version.
- Enter the reconciled forward translation in column *Reconciled Forward Translation Of Translators*.
- Note the reasons why the reconciled item translation is the best equivalent to the English source in column *Comments*.

### Guidelines for the reconciliation of the forward translations from English into target language by experts

- Review the forward translations for each item.
- Select one of the forward translations for each item or provide a new option; this could be a modified version of one of the forward translations, a hybrid version, or a new version.
- Fill in the reconciled forward translation in column *Reconciled Universal Forward Translation*.
- Note the reasons why the reconciled item translation is the best equivalent to the English source in column *Comments*.

Please pay close attention to following rules:

- Child-friendly wording.
- The item context is not part of the item sentence. Context and stem have to be translated as two separated Sects. (1. Stem, 2. Context), so the time information in the context would be replaceable (e.g., “In the past 4 weeks” > “In the past 3 months”).
- In order to maintain the context and stem separate, present the context followed by a colon (e.g., “In the past 4 weeks: Thinking about…”).
- For culture-specific words (e.g. mile, blocks, football) find a conceptually equivalent translation (e.g., mile > km).
- Translate the items conceptually equivalent, even if the same word has to be chosen for different (more or less synonymous) original words (e.g., afraid, anxious, scared, frightened, fearful > “ängstlich”). For a precise wording note the intended intensity and direction of the original word.
- The items can be translated in a different grammatical tense, if this contributes to a better understanding of the item (e.g., imperfect instead of perfect, or vice versa).

### Guidelines for Back-translation (BT) into English

- The translator should only be provided with the reconciled version of the target language translation. The translator should not have either of the original forward translations or the original English source questionnaire.
- The BT into English is not merely a literal translation of the translated phrase but must reflect what the target language translation says, without embellishing it. A conceptual BT is required.
- Keep in mind that the BT into English is not the final product. Its purpose is to help assess the accuracy of the reconciled version of the target language translation.
- The BT includes such information as whether there are alternative meanings or connotations and/or any culture-specific issues that may be construed in the translated phrase.
- Fill in the BT of the response options as well as the item stems (spreadsheets *ANG, ANX, DEP, FAT, PAIN, PEER*) in column *Backward Translation*.
- Notes regarding the BT can be documented in column *Comments*.

### Guidelines for review of the back-translation (BT) and finalization

- Review the translation history for each item.
- Review the source item, the reconciled version of the target language translation, the English language BT of the item in the target language, and all comments and suggestions made by reconciler(s).
- Enter your suggested final translation of the response options and item-stems (*ANG, ANX, DEP, FAT, PAIN, PEER*) in column *Final Translation*.
- Address and respond to comments along with explanation for your choice of final translation in column *Comments*.
- Provide BT of your translation suggestion if different from reconciled version.

After the review of the BTs is completed, it’s up to the reviewers of the target language to make a recommendation on the final translation:

- Comments that deal with the meaning of an item or with the apparent overlap between items require feedback from the developers before deciding if the target language translation is correct.
- If the BT differs from the source item in a meaningful way, it’s either because the BT is wrong or because the target language translation is inaccurate. It’s up to the language-specific reviewers to either confirm the accuracy of their own language translation or correct it.
- It is also possible that the BT matches the English source item, but the language-specific reviewers identify a problem in their language translation. Again, the issue or discrepancy should be documented and the German translation for that item should be adjusted as needed.
- If the reviewers for each language suggest different resolutions for the issues identified during the BT review, the Language Coordinator will make a decision (of course aiming for a universal translation as much as possible).

### Guidelines for Finalization of the test version of the translation

*According to the FACIT (Functional Assessment of Cancer Therapy)-Translation Methodology this step is performed by a language coordinator who is a native speaker of the target language, usually one of the reviewers or the reconciler. The final translation is suggested to be the product of an agreement between the reviewers, who are native speakers of the target language.*

- Review the source and all of the reviewers’ comments and suggestions.
- Provide the final translation. Fill in the final versions in column “Final Universal Translation”.
- Explain your choice if the final translation is different from the reconciled version or from what reviewers recommended individually in column “comments”.

## Appendix 3: Translated item banks/short forms:

PROMIS Pediatric SF v1.1 – Anger (5 items)—PROMIS Parent Proxy SF v1.0 – Anger (5 items).

PROMIS Pediatric Bank v1.1 – Anxiety (13 items)—PROMIS Parent Proxy Item Bank v1.1 – Anxiety (13 items).

PROMIS Pediatric Bank v1.1 – Depressive Symptoms (13 items)—PROMIS Parent Proxy Item Bank v1.1 – Depressive Symptoms (13 items).

PROMIS Pediatric Bank v1.0 – Fatigue Tired (23 items)—PROMIS Parent Proxy Item Bank v1.0 – Fatigue Tired (23 items).

PROMIS Pediatric Bank v1.0 – Pain Interference (13 items)—PROMIS Parent Proxy Item Bank v1.0 – Pain Interference (13 items).

PROMIS Pediatric Bank v1.0 – Peer Relationships (15 items)—PROMIS Parent Proxy Item Bank v1.0 – Peer Relationships (15 items).

Exemplary self-report items of the German translation version of the Pediatric PROMIS item banks only – due to copyright restrictions. Please see the complete item versions at https://www.healthmeasures.net/search-view-measures..

**Table:**

Item code	Measure (SF: short form; IB: item bank)	Original English item	Final German translation
PROMIS Anger: 2 out of 5 items		In the past 7 days,	In den letzten 7 Tagen,
206R1	SF V2.0, IB V.1.1	I felt mad	Ich war wütend
2319aR1	SF V2.0, IB V.1.1	I was so angry I felt like throwing something	Ich war so wütend, dass ich etwas herumschmeißen wollte
PROMIS Anxiety: 3 out of 13 items		In the past 7 days,	In den letzten 7 Tagen,
713R1	SF V2.0, IB V2.0, IB V.1.1	I felt nervous	Ich war nervös
227bR1	SF V2.0, IB V2.0, IB V.1.1	I felt scared	Ich habe mich gefürchtet
231R1	SF V2.0, IB V2.0, IB V.1.1	I worried about what could happen to me	Ich habe mir Sorgen darüber gemacht, was mir zustoßen könnte
PROMIS Pediatric Depressive Symptoms: 3 out of 13 items		In the past 7 days,	In den letzten 7 Tagen,
488R1	SF V2.0, IB V2.0, IB V.1.1	I could not stop feeling sad	Ich konnte nicht aufhören, traurig zu sein
711R1	SF V2.0, IB V2.0, IB V.1.1	I felt lonely	Ich fühlte mich allein
3952aR2	SF V2.0, IB V2.0, IB V.1.1	It was hard for me to have fun	Es ist mir schwergefallen, Spaß zu haben
PROMIS Pediatric Fatigue: 5 out of 23 items		In the past 7 days,	In den letzten 7 Tagen,
4239aR2	SF V2.0, IB V2.0, IB V.1.0	Being tired made it hard for me to keep up with my schoolwork	Weil ich müde war, ist es mir schwergefallen, mit den Aufgaben in der Schule und Hausaufgaben hinterherzukommen
2876R1	SF V2.0, IB V2.0, IB V.1.0	I got tired easily	Ich bin schnell müde geworden
4241R2	SF V2.0, IB V2.0, IB V.1.0	I was too tired to do sports or exercise	Ich war zu müde für Sport oder Bewegung
3548R1	IB V2.0, IB V.1.0	I felt too tired to spend time with my friends	Ich war zu müde, um Zeit mit meinen Freunden zu verbringen
4194R1	IB V2.0, IB V.1.0	I needed to sleep during the day	Ich habe tagsüber Schlaf gebraucht
PROMIS Pain Interference: 3 out of 13 items		In the past 7 days,	In den letzten 7 Tagen,
2049R1	SF V2.0, IB V2.0, IB V.1.0	It was hard for me to walk one block when I had pain	Es ist mir schwergefallen, eine kurze Strecke (100 m) zu Fuß zu gehen, wenn ich Schmerzen hatte
3582R1	IB V2.0, IB V.1.0	I hurt a lot	Ich hatte starke Schmerzen
1701R1	IB V2.0, IB V.1.0	It was hard to get along with other people when I had pain	Es ist mir schwergefallen, gut mit anderen auszukommen, wenn ich Schmerzen hatte
PROMIS Pediatric Peer Relationships: 4 out ot 15 items		In the past 7 days,	In den letzten 7 Tagen,
5018R1	SF V2.0, IB V2.0, IB V.1.0	I felt accepted by other kids my age	Ich fühlte mich von anderen Kindern/Jugendlichen in meinem Alter akzeptiert
5058R1	SF V2.0, IB V2.0, IB V.1.0	I was able to count on my friends	Ich konnte auf meine Freunde zählen
5056R1	SF V2.0, IB V2.0, IB V.1.0	I was able to talk about everything with my friends	Ich konnte mit meinen Freunden über alles reden
5055R1	SF V2.0, IB V2.0, IB V.1.0	My friends and I helped each other out	Meine Freunde und ich haben uns gegenseitig geholfen

Exemplary proxy-report items of the German translation version of the Pediatric PROMIS item banks only – due to copyright restrictions. Please see the complete item versions at https://www.healthmeasures.net/search-view-measures..

**Table:**

Item code	Measure	Original English item	Final German translation
PROMIS Anger: 2 out of 5 items		In the past 7 days,	In den letzten 7 Tagen,
Pf1anger1	SF V2.0, IB V.1.0	My child felt mad	Mein Kind war wütend
Pf1anger3	SF V2.0, IB V.1.0	My child was so angry he/she felt like throwing something	Mein Kind war so wütend, dass es etwas herumschmeißen wollte
PROMIS Anxiety: 3 out of 13 items		In the past 7 days,	In den letzten 7 Tagen,
Pf1anxiety8	SF V2.0, IB V2.0, IB V.1.1	My child felt nervous	Mein Kind war nervös
Pf2anxiety2	SF V2.0, IB V2.0, IB V.1.1	My child felt scared	Mein Kind hat sich gefürchtet
Pf1anxiety3	SF V2.0, IB V2.0, IB V.1.1	My child worried about what could happen to him/her	Mein Kind hat sich Sorgen darüber gemacht, was ihm zustoßen (zustossen) könnte
PROMIS Pediatric Depressive Symptoms: 3 out of 13 items		In the past 7 days,	In den letzten 7 Tagen,
Pf2depr7	SF V2.0, IB V2.0, IB V.1.1	My child could not stop feeling sad	Mein Kind konnte nicht aufhören, traurig zu sein
Pf2depr10	SF V2.0, IB V2.0, IB V.1.1	My child felt lonely	Mein Kind fühlte sich einsam
Pf2depr6	SF V2.0, IB V2.0, IB V.1.1	It was hard for my child to have fun	Es ist meinem Kind schwergefallen, Spaß/Spass zu haben
PROMIS Pediatric Fatigue: 5 out of 23 items		In the past 7 days,	In den letzten 7 Tagen,
Pf2fatigue8	SF V2.0, IB V2.0, IB V.1.0	Being tired made it hard for my child to keep up with schoolwork	Weil mein Kind müde war, ist es ihm schwergefallen, mit seinen Schul- und Hausaufgaben hinterherzukommen
Pf4fatigue3	SF V2.0, IB V2.0, IB V.1.0	My child got tired easily	Mein Kind ist leicht müde geworden
Pf3fatigue8	SF V2.0, IB V2.0, IB V.1.0	My child was too tired to do sports or exercise	Mein Kind war zu müde für Sport oder Bewegung
Pf2fatigue7	IB V2.0, IB V.1.0	My child felt too tired to spend time with his/her friends	Mein Kind war zu müde, um Zeit mit seinen Freunden zu verbringen
Pf1fatigue5	IB V2.0, IB V.1.0	My child needed to sleep during the day	Mein Kind hat tagsüber Schlaf gebraucht
PROMIS Pain Interference: 3 out of 13 items		In the past 7 days,	In den letzten 7 Tagen,
Pf1pain4	SF V2.0, IB V2.0, IB V.1.0	It was hard for my child to walk one block when he/she had pain	Es ist meinem Kind schwergefallen, eine kurze Strecke (100 m) zu Fuß (Fuss) zu gehen, wenn es Schmerzen hatte
Pf4pain1	IB V2.0, IB V.1.0	My child hurt a lot	Mein Kind hatte starke Schmerzen
Pf4pain4	IB V2.0, IB V.1.0	It was hard for my child to get along with other people when he/she had pain	Es ist meinem Kind schwergefallen, mit anderen auszukommen, wenn es Schmerzen hatte
PROMIS Pediatric Peer Relationships: 4 out of 15 items		In the past 7 days,	In den letzten 7 Tagen,
Pf3socabil9	SF V2.0, IB V2.0, IB V.1.0	My child felt accepted by other kids his/her age	Mein Kind fühlte sich von anderen Kindern/Jugendlichen in seinem Alter akzeptiert
Pf4socabil12	SF V2.0, IB V2.0, IB V.1.0	My child was able to count on his/her friends	Mein Kind konnte auf seine Freunde zählen
Pf4socabil10	IB V2.0, IB V.1.0	My child was able to talk about everything with his/her friends	Mein Kind konnte mit seinen Freunden über alles reden
Pf2socrole4	SF V2.0, IB V2.0, IB V.1.0	My child and his/her friends helped each other out	Mein Kind und seine Freunde haben sich gegenseitig geholfen

Items are displayed with 5 point response options.

## Abbreviations

ANG: Anger
ANX: Anxiety
BELLA: BEfragung zum seeLischen WohLbefinden und VerhAlten
BT: Backward translation
CAT: Computerized adaptive test
CI: Cognitive interview
CUFT: Consented universal forward translation
CUT: Consented universal translation
DEP: Depression
FACIT: Functional assessment of chronic illness therapy
FAT: Fatigue
FT: Forward translation
IRT: Item response theory
ISPOR: International society for pharmacoeconomics and outcomes
P: Pain
PR: Peer relationships
PRO: Patient reported outcome
PROMIS^®^: Patient reported outcome measurement information system
RFT: Reconciled forward translation
RUFT: Reconciled universal forward translation
TPM: Translation process manager
